# The histone 3 lysine 9 methyltransferase inhibitor chaetocin improves prognosis in a rat model of high salt diet-induced heart failure

**DOI:** 10.1038/srep39752

**Published:** 2017-01-04

**Authors:** Tomohiko Ono, Naomi Kamimura, Tomohiro Matsuhashi, Toshihiro Nagai, Takahiko Nishiyama, Jin Endo, Takako Hishiki, Tsuyoshi Nakanishi, Noriaki Shimizu, Hirotoshi Tanaka, Shigeo Ohta, Makoto Suematsu, Masaki Ieda, Motoaki Sano, Keiichi Fukuda, Ruri Kaneda

**Affiliations:** 1 Department of Cardiology, Keio University School of Medicine, Shinjuku-ku Tokyo, Japan; 2 Department of Biochemistry and Cell Biology, Institute of Development and Aging Sciences, Graduate School of Medicine, Nippon Medical School, Kawasaki, Kanagawa, Japan; 3 Electron Microscope Laboratory, Keio University Hospital, Shinjuku-ku, Tokyo, Japan; 4 Department of Biochemistry, Keio University School of Medicine, Shinjuku-ku, Tokyo, Japan; 5 MS Business Unit, Shimadzu Corporation, Kyoto, Japan; 6 Division of Rheumatology, Center for Antibody and Vaccine Therapy, IMSUT Hospital, The Institute of Medical Science, The University of Tokyo, Minato-ku, Tokyo, Japan; 7 Division of Anti-aging Medicine, Center for Molecular Medicine, Jichi Medical University, Shimotsukeshi, Tochigi, Japan; 8 JST, PRESTO, Kawaguchi, Saitama, Japan; 9 Clinical and Translational Research Center, Keio University School of Medicine, Shinjuku-ku, Tokyo, Japan

## Abstract

Histone acetylation has been linked to cardiac hypertrophy and heart failure. However, the pathological implications of changes in histone methylation and the effects of interventions with histone methyltransferase inhibitors for heart failure have not been fully clarified. Here, we focused on H3K9me3 status in the heart and investigated the effects of the histone H3K9 methyltransferase inhibitor chaetocin on prognoses in Dahl salt-sensitive rats, an animal model of chronic heart failure. Chaetocin prolonged survival and restored mitochondrial dysfunction. ChIP-seq analysis demonstrated that chronic stress to the heart induced H3K9me3 elevation in thousands of repetitive elements, including intronic regions of mitochondria-related genes, such as the gene encoding *peroxisome proliferator-activated receptor-gamma coactivator 1 alpha*. Furthermore, chaetocin reversed this effect on these repetitive loci. These data suggested that excessive heterochromatinization of repetitive elements of mitochondrial genes in the failing heart may lead to the silencing of genes and impair heart function. Thus, chaetocin may be a potential therapeutic agent for chronic heart failure.

Heart failure is one of the leading causes of death worldwide^1^,^2^. Although existing heart failure therapies that target the renin-angiotensin-aldosterone and adrenergic nervous systems are effective^3^,^4^,^5^,^6^,^7^, mortality from heart failure remains high^1^,^2^,^8^. Novel insights into the mechanisms underlying this disease are necessary to establish a more effective therapeutic strategy. Mitochondria and metabolic function have been targeted for the treatment of heart failure because they are essential for myocardial energy production, cell redox potential, reactive oxygen species (ROS) generation, mitochondria-dependent apoptosis, calcium homeostasis, and fatty acid and glucose metabolism.

Pathological hypertrophy and heart failure are associated with altered expression of a number of genes^9^,^10^,^11^,^12^,^13^. Asakura *et al*. reported that many heart failure-related genes are involved in the pathways of mitochondrial function, oxidative phosphorylation, and extracellular signaling^14^. Epigenetic alterations play an important role in the regulation of transcriptional activity. Histone modifications can alter chromatin structure to influence transcription factor access to the DNA and the recruitment of transcriptional complexes to gene promoter/enhancer regions^15^. Histone acetylation and deacetylation are known to play a role in the development of cardiac hypertrophy and heart failure^16^,^17^. Furthermore, inhibition of histone deacetylase (HDAC) activity prevents cardiac remodeling^18^. HDAC inhibitors are likely to have multiple mechanisms of efficacy^19^, including inhibition of cardiac hypertrophy^20^,^21^, autophagy^22^, apoptosis^23^, cardiac fibrosis^24^,^25^, inflammation^26^,^27^, and regulation of cardiac contractility^28^.

We have previously shown that the chromosomal distributions of histone H3 lysine 9 trimethylation (H3K9me3) and histone H3 lysine 4 trimethylation (H3K4me3) rather than that of histone acetylation differ between the normal and failed heart in animal models of heart failure^29^. Additional evidence suggests that histone methylation on lysine 4 (K4), K9, or K36 of histone H3 are involved in cardiac remodeling^30^,^31^,^32^,^33^. Therefore, both histone methylation and histone acetylation may act as therapeutic targets for the management of heart failure.

H3K9me3 is associated with heterochromatin formation and is required to establish the pericentromere and telomere regions^34^. Alterations in H3K9me3 levels at various genomic loci, including satellite repeats and repetitive transposable elements, are also associated with cancer and stress responses^35^,^36^. However, neither the role of H3K9me3 on repetitive loci in the failing heart nor the efficacy of histone methyltransferase inhibitors in heart failure has been clarified.

Therefore, in this study, we investigated H3K9 trimethylation status on repetitive elements in the failing heart and hypothesized that the histone-modifying enzyme affecting H3K9 trimethylation status may play an important role in heart failure. SU(VAR)3-9 is an enzyme that catalyzes the conversion of K9 in histone H3 from a dimethylated form to a trimethylated form. Chaetocin, a natural small molecule produced by fungi of the *Chaetomium* species^37^, is an inhibitor of SU(VAR)3-9^38^. Thus, we investigated whether this H3K9 methyltransferase inhibitor blocked the progression of heart failure in an animal model. In this study, we showed that chaetocin delayed the transition from hypertrophy to heart failure, restored mitochondrial dysfunction in failing hearts, and prolonged animal survival.

## Results

### Chaetocin improved the prognosis of Dahl salt-sensitive (DS) rats with heart failure

**Figure 1:**
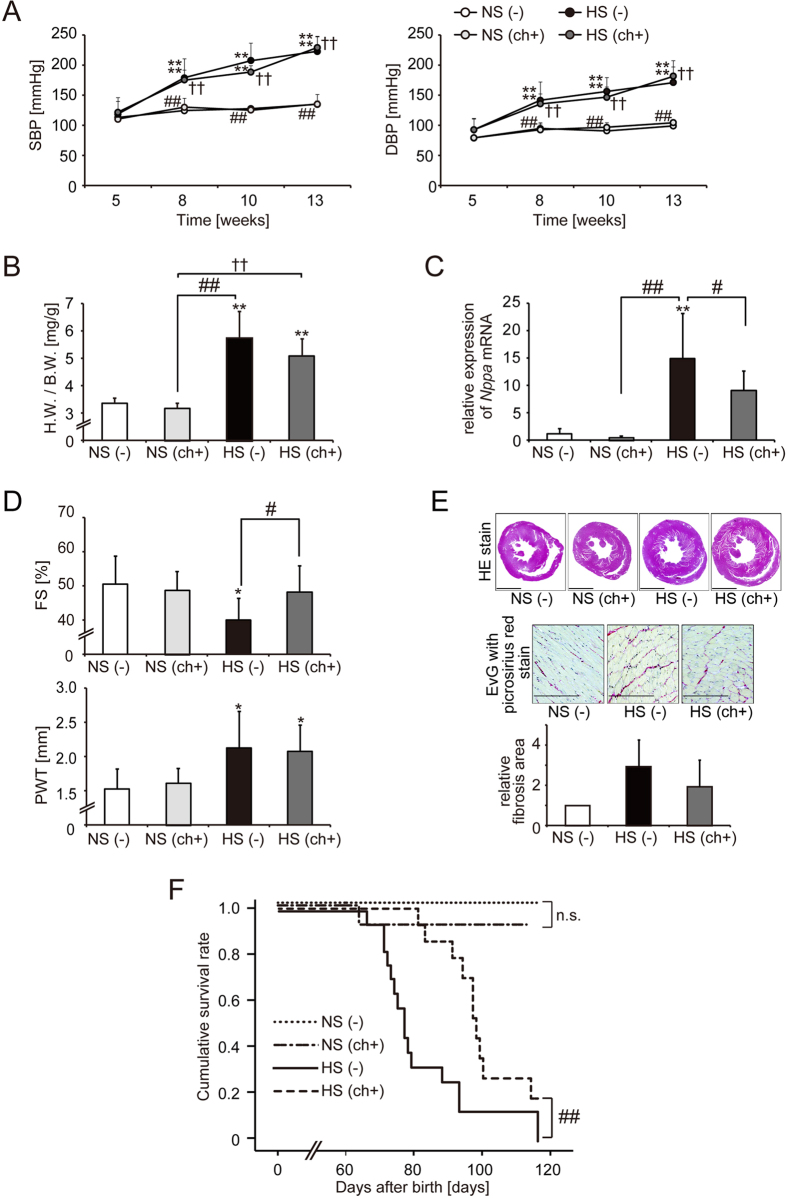
Chaetocin improved the prognosis of rats in a model of heart failure. (**A**) Systolic and diastolic blood pressures (SBP and DBP, respectively). (**B**) Heart weight/body weight (HW/BW) ratios are shown for 13-week-old rats in the control group [NS (−), n = 11], normal salt diet with chaetocin group [NS (ch+), n = 7], HF group [HS (−), n = 17], and treatment group [HS (ch+), n = 17]. (**C**) Quantitative real-time PCR analysis of *natriuretic peptide precursor A (Nppa, ANP*) mRNA expression in left ventricular tissues for 13-week-old rats in the control group (n = 5), normal salt diet with chaetocin group (n = 4), HF group (n = 10), and treatment group (n = 14). (**D**) Echocardiographic measurements of fractional shortening (FS) and posterior wall thickness (PWT). (**E**) Hematoxylin-eosin-stained (upper panel, scale bars: 2 mm) or Elastica van Gieson (EvG) with picrosirius red-stained sections of cardiac tissue (middle panel, scale bars: 200 μm) and quantification of the fibrotic area (lower bar). (**F**) Kaplan-Meier survival analysis of DS rats in the control group (n = 9), normal salt diet with chaetocin group (n = 12), HF group (n = 17), and treatment group (n = 13). Data are presented as the mean ± SD. *P < 0.05 and **P < 0.01 versus the control group; ^#^*P* < 0.05 and ^##^*P* < 0.01 versus the HF group; ^†^*P* < 0.05 and ^††^*P* < 0.01 versus the normal salt diet with chaetocin group; n.s.: not significant.

A high-salt diet in DS rats induced hypertension, leading to left ventricular (LV) hypertrophy and heart failure at around 13 weeks. The blood pressures of animals consuming a high-salt diet were high, regardless of chaetocin administration (Fig. 1A). At the age of 13 weeks, the body weights of animals fed a high-salt diet were lower than those of animals fed a normal-salt diet (Supplemental Fig. 1). Heart weight/body weight (HW/BW) ratios were higher in the heart failure (HF) group [high-salt diet containing 8% NaCl, HS (−)] than in the control group [normal-salt diet containing 0.3% NaCl, NS (−)]. The increases in HW/BW ratios in rats consuming the high-salt diet were not significantly reversed by administration of chaetocin [high-salt diet with 0.25 mg/kg of chaetocin, HS (ch+); Fig. 1B]. The expression of the gene encoding *natriuretic peptide precursor A (Nppa*), a marker of heart failure, was upregulated in LV tissues in the HF group, and this increase in expression was significantly suppressed by chaetocin administration (Fig. 1C). To determine whether chaetocin could improve cardiac function, we performed echocardiographic studies in 13-week-old rats. LV systolic function, represented by fractional shortening (FS), was significantly improved following treatment with chaetocin. Additionally, LV posterior wall thickness was not changed following chaetocin treatment (Fig. 1D). These data indicated that chaetocin did not affect the development of cardiac hypertrophy, but did maintain LV systolic function. To assess the influence of chaetocin on cardiac fibrosis, we examined cardiac tissues microscopically. Fibrotic areas were detected using Elastica van Gieson (EvG) stain with picrosirius red staining of sectioned tissue. The fibrotic area tended to be larger in the HF group than in the control group, and administration of chaetocin tended to reduce cardiac fibrosis (Fig. 1E). The survival rate was significantly improved by treatment with chaetocin (Fig. 1F). These data suggested that chaetocin improved the prognosis of DS rats with heart failure by delaying the transition from hypertrophy to heart failure.

### Chaetocin restored the downregulation of mitochondria-related genes in heart failure

**Figure 2:**
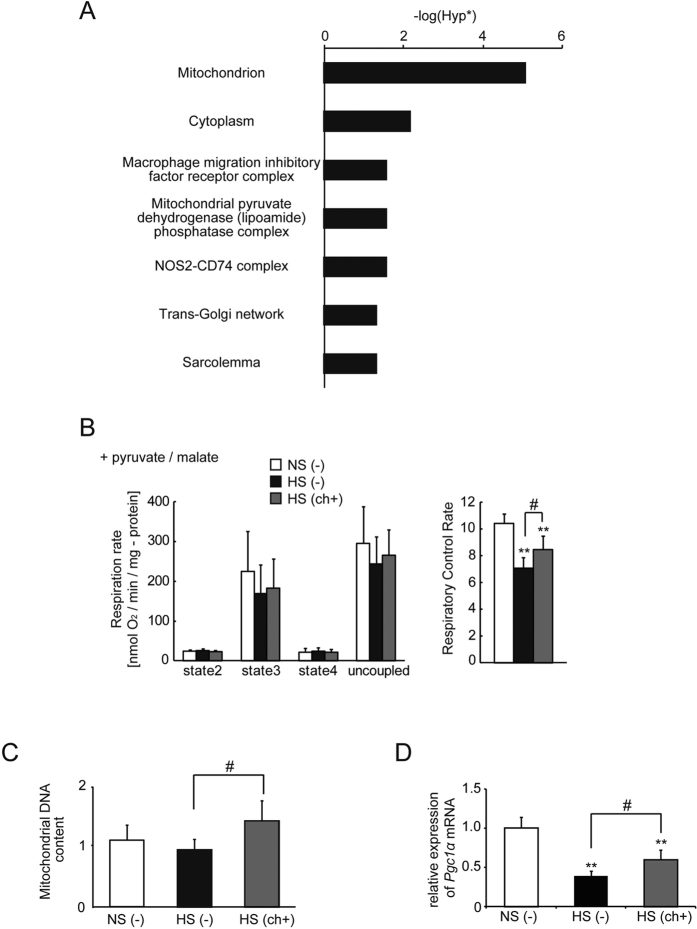
Gene ontology analysis of the set of 50 genes that were downregulated in failing hearts and restored with chaetocin; restoration of mitochondrial function with chaetocin. (**A**) Singular enrichment analysis of the “cellular component” category. Hyp* means *p*-value calculated using the corrected hypergeometric distribution. (**B**) Mitochondrial respiration (each group, n = 5). Substrates: pyruvate/malate. (**C**) Mitochondrial DNA content (control group [n = 5], HF group [n = 5], and treatment group [n = 7]). (**D**) Quantitative real-time PCR analysis of peroxisome proliferator-activated receptor gamma, coactivator 1 alpha (*Pgc1α*) mRNA expression in left ventricular tissues (each group, n = 4) at 13 weeks of age. Data are presented as the mean ± SD. **P < 0.01 versus the control group; ^#^*P* < 0.05 versus the HF group.

To determine the mechanisms underlying the effects of chaetocin, we used DNA microarray analysis in LV tissues of DS rats. Chaetocin is an inhibitor of SU(VAR)3-9 methyltransferase activity^38^. Therefore, we focused on genes whose expression levels decreased in failing hearts and were restored with chaetocin treatment. Fifty genes were identified based on the following criteria: (1) the ratio of gene expression in the HF group to that in the control group was less than 0.5; (2) the ratio of gene expression in the treatment group to that in the HF group was greater than 1.4; (3) the normalized expression level in the HF group was equal to or greater than 100 (Table 1). “Mitochondrion” was identified as the gene ontology (GO) term with the smallest Hyp* value by singular enrichment analysis of the “cellular component” category in the GeneCodis3 database (http://genecodis.cnb.csic.es/release/) using the set of 50 genes shown in Table 1 (Fig. 2A). Therefore, we focused on mitochondrial function. Detailed results for the GO analyses of the 50 identified genes are given in Supplemental Tables 3 and 4.

### Chaetocin improved mitochondrial respiration and increased the mitochondrial content in failing hearts

To investigate whether chaetocin improved mitochondrial function, we measured mitochondrial respiration with pyruvate/malate as an energy source. Respiration states (2–4) and uncoupled respiration rates were calculated as nmol of oxygen/min/mg of mitochondrial protein. State 3 and uncoupled respiration of mitochondria isolated from rats in the HS (ch+) group tended to be higher than that from rats in the HS (−) group, although statistical significance was never reached (p > 0.1). Importantly, in every mitochondrial preparation, a small amount of contaminants from other organelles and debris may be present and contribute to the overall protein concentration. Moreover, mitochondrial preparations from damaged tissue may be unstable and cause variability in the data when the same amount of protein is used for each respiration assay. Respiratory control rates (RCRs), an index of the overall health of the mitochondria, are calculated from the ratio of state 3 to state 4 and the RCR value is independent of preparation variability. Consequently, a significant increase in the RCR value was found in rats in the HF with chaetocin group [HS (ch+)] (Fig. 2B).

Although the mitochondrial DNA content was not different between the control group and HF group, a significant increase in mitochondrial DNA content was observed following treatment with chaetocin (Fig. 2C). Moreover, the expression of the gene encoding *peroxisome proliferator-activated receptor gamma, coactivator 1 alpha (Pgc1α*), which plays important roles in mitochondria biogenesis and energy production, was significantly increased after chaetocin administration (Fig. 2D). These findings suggested that chaetocin improved mitochondrial respiration mediated by the increase in mitochondrial DNA content and expression of *Pgc1α* in failing hearts.

### Heart failure increased H3K9me3 levels on repetitive elements, and this effect was reversed following chaetocin treatment

**Figure 3:**
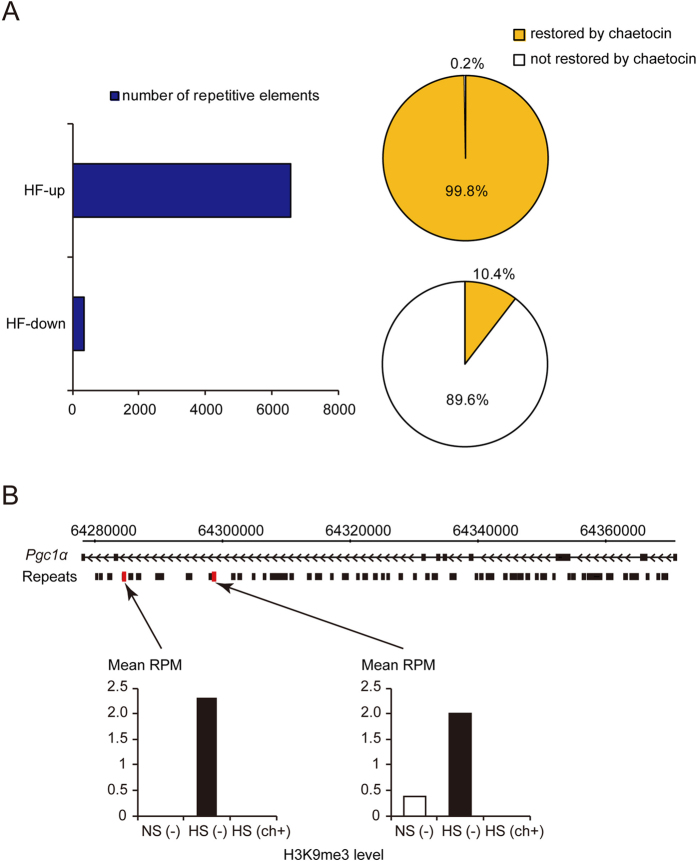
Heart failure increased H3K9me3 levels on repetitive elements. (**A**) Number of repetitive elements in which heart failure caused an increase (HF-up) or decrease (HF-down) in H3K9me3 compared with control samples (left). The restoration percentage of H3K9 trimethylation state following treatment with chaetocin (right). (**B**) H3K9me3 levels in the intronic repetitive regions of *Pgc1α*. Red squares indicate the region that was identified as being enriched in H3K9me3 repetitive elements in rats with heart failure. The black boxes indicate the repetitive loci. The bars indicate the H3K9me3 read alignments of the repetitive elements. RPM, reads per million mapped.

To investigate H3K9me3 status in the whole genome, including repetitive elements in the heart, we performed chromatin immunoprecipitation (ChIP) for analysis of sequences exhibiting H3K9me3 in the failed LV with or without chaetocin treatment and in controls. At 6550 loci associated with repetitive elements, heart failure caused an increase in H3K9me3 alignment compared with that in control samples. We defined these elements as “HF-up.” Ninety-nine percent of HF-up loci, i.e., 6534 repetitive elements, showed a corresponding reduction in H3K9me3 in response to chaetocin treatment. In contrast, at 335 loci, we observed a reduction in H3K9me3 alignment in the failing heart compared with that in the controls. We defined these elements as “HF-down.” Administration of the inhibitor reversed this effect for 10.4% of these HF-down loci, i.e., 35 repetitive elements (Fig. 3A). Thus, HF increased H3K9me3 levels on repetitive elements, and chaetocin altered H3K9me3 levels in those loci, as expected based on the inhibitory activity of H3K9 methyltransferase in heart tissues.

### Chaetocin reduced H3K9me3 levels on intronic repetitive elements of Pgc1α

**Figure 4:**
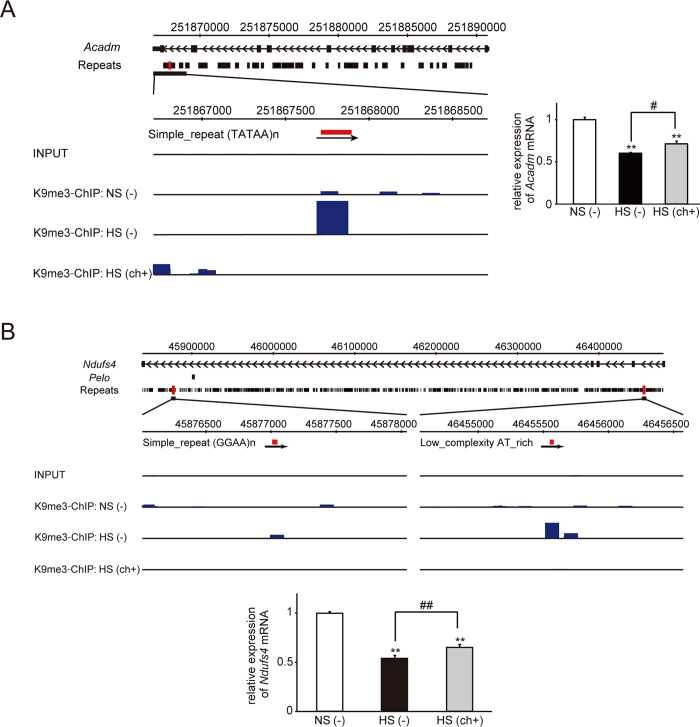
Representative data showing enrichment of H3K9me3 repetitive loci in regions in close proximity to *Acadm* (**A**) and *Ndufs4* (**B**) in the HF group. Red squares indicate the region that was identified as being enriched in H3K9me3 repetitive elements in rats with heart failure. The black boxes indicate the repetitive loci. The blue bars indicate H3K9me3 read alignments. The mRNA level of each gene was determined with the real-time quantitative PCR and is shown as the fold change versus the control group. **P < 0.01 versus the control group; ^#^*P* < 0.05 and ^##^*P* < 0.01 versus the HF group.

In this study, we focused on genomic regions in close proximity to RefSeq genes. Two repetitive loci in the intron of *Pgc1α* exhibited elevated H3K9me3 levels in the failing heart, and this effect was suppressed by chaetocin treatment (Fig. 3B). The other repetitive loci exhibiting elevated H3K9me3 levels in the failing heart included several genomic regions located in close proximity to mitochondrial genes. For example, we identified the following gene regions: an intron of Acyl-CoA dehydrogenase, medium-chain, *Acadm* (Fig. 4A); two intronic regions of NADH-ubiquinone oxidoreductase Fe-S protein 4, *Ndufs4* (Fig. 4B); and a region in an intron for hexaprenyldihydroxybenzoate methyltransferase, mitochondrial precursor, *Coq3* (Supplemental Fig. 2). Consistent with the H3K9me3 epigenetic profile, the mRNA levels of *Acadm* and *Ndufs4* were reciprocally lowest in the failing heart, as judged from quantitative real-time PCR. Although the restoration of *Coq3* mRNA with treatment was not significant, elevated H3K9me3 in the failing heart compared with that in the control heart may have contributed to down-regulation of *Coq3* expression.

### GO analyses of “HF-up” H3K9me3 repetitive loci reversed by chaetocin

The ratio of the length of repetitive sequences to the genomic length of whole RefSeq genes, including 10 kb up- or down-stream of those genes, was 31.905%. Additionally, the ratio of the genomic regions for genes categorized as “mitochondrion” was 31.791%. These data suggested that the length of repetitive sequences relative to the genome was not different between whole RefSeq genes and genes categorized as “mitochondrion”. A total of 2588 regions identified as showing the “HF-up” H3K9me3 loci reversed by chaetocin on any RefSeq gene or within 10 kb up- or down-stream of those genes were registered within GeneCodis3. Of those regions, the number of genes categorized as “mitochondrion” was 161 (6.2%). The number of whole RefSeq genes of the rat genome on GeneCodis3 was 29516. Of these genes, the number of genes categorized as “mitochondrion” by GO analysis was 1247 (4.2%). Therefore, enrichment of the “HF-up” H3K9me3 repetitive loci reversed by chaetocin to the GO “mitochondrion” was 1.47-fold greater than that observed by chance. The Hyp* value was 0.00000651241. These regions of the “HF-up” H3K9me3 repetitive loci reversed by chaetocin included the intronic regions of *Acadm, Ndufs4*, and *Coq3*.

In conclusion, these data suggested that chronic stress to the heart caused excessive heterochromatinization on repetitive elements, including regions neighboring mitochondrial genes, such as *Pgc1α*, thereby decreasing the expression of the target genes and leading to mitochondrial dysfunction. H3K9 methyltransferase inhibitors may have promising applications as novel therapies for chronic heart failure by reducing excess heterochromatinization at repetitive regions in the genome and restoring heart function.

## Discussion

In this study, we demonstrated that the histone H3K9 methyltransferase inhibitor chaetocin blocked the progression of heart failure and alleviated mitochondrial dysfunction. This epigenetic treatment may improve outcomes in patients with heart failure.

Alterations in H3K9me3 levels at various genomic loci, including satellite repeats and repetitive transposable elements, have been shown to be associated with cancer and stress responses^35^,^36^; therefore, we focused on the H3K9me3 status of repetitive elements. Hunter *et al*. reported that “acute” stress increases H3K9me3 levels of a transposable element and that H3K9me3 suppresses the stress-induced activation of repetitive and transposable elements as a protective response^36^. In contrast, Chan *et al*. found that GAA-triplet intronic expansions stimulate heterochromatinization and cause transcriptional repression of the Frataxin gene, leading to Friedreich’s ataxia^39^. Furthermore, Asano *et al*. suggested that products of intron-processed retroposons of the *laminin receptor 1* gene interact with heterochromatin protein 1 (HP1) to cause degeneration of cardiomyocytes^40^. Consistent with a previous study, stress to the heart may have induced H3K9 methylation of repetitive elements in intronic regions in our study. Based on the previous two reports^39^,^40^, heterochromatinization of repetitive elements around critical genes or the formation of heterochromatin with HP1 may contribute to the pathophysiology of chronic heart failure. The *Pgc1α* gene, which we identified as “HF-up” and was found to exhibit restored H3K9me3 levels after chaetocin treatment, plays a key role in mitochondrial biogenesis and energy production. In addition to *Pgc1α*, we identified other genes that were related to mitochondrial function in this study. *Acadm* encodes an enzyme that catalyzes the initial reaction in the beta-oxidation of fatty acids^41^. The protein product of *Coq3* is a critical component of the electron transport pathways in both eukaryotes and prokaryotes^42^. *Ndufs4* encodes a component of the first multi-subunit enzyme complex of the mitochondrial respiratory chain^43^, which plays a vital role in cellular ATP production. Compared with the level of restoration of *Pgc1α* gene expression, the improvement in cardiac systolic function was more drastic. Therefore, the parallel restoration of the expression of these mitochondria-function related genes, i.e., *Acadm* and *Ndufs4*, may have caused this effect.

Furthermore, we speculate that the discrepancy between the complete change in H3K9me3 in response to chaetocin in the repetitive elements of *Pgc1α* and the smaller restoration of *Pgc1α* gene expression may be explained by the occurrence of H3K9me3 and other epigenetic modifications during the progression and development of hypertrophy/heart failure. For example, histone deacetylation and DNA methylation at the promoter region may have occurred. Additional administration of the HDAC inhibitor may be more effective at restoring mRNA expression to basal levels.

Our results suggested that chronic stress to the heart may gradually promote excessive heterochromatinization at repeats in the introns of critical genes for pumping function, such as genes related to mitochondrial function. The H3K9 methyltransferase inhibitor chaetocin may maintain the appropriate chromatin structure and reverse the excessive heterochromatinization. Therefore, histone H3K9 methyltransferase inhibitors may represent potential novel therapies for chronic heart failure. Moreover, although chaetocin was initially identified as the first specific inhibitor of SU(VAR)3-9^38^, a recent report from another laboratory showed that it may not exhibit high specificity^44^. The disulfide bridge of the epipolythiodioxopiperazine (ETP) unit may have a central role in mediating the histone lysine methyltransferase inhibitory activity of chaetocin through a nonspecific mechanism. Other ETP natural products can also inhibit histone lysine methyltransferases^45^. Thus, it will be important to investigate whether other ETP products have similar effects on improving the prognosis of heart failure. Moreover, further experiments are needed to determine which histone lysine methyltransferase activity is inhibited by chaetocin in this model. In addition, as we cannot exclude the possibility that improvement of cardiac function by chaetocin caused the restoration of mitochondrial function-related gene expression, additional experiments using techniques, such as knockdown of Su(var)3–9, are required in order to show that alterations of H3K9me3 level or those of expression in mitochondrial function-related genes are primary.

## Materials and Methods

### Animals and drug delivery

All animal experiments in this study were approved by the Keio University Institutional Animal Care and Use Committee (09088) and were carried out in accordance with the Institutional Guidelines on Animal Experimentation at Keio University. Five-week-old male DS rats were purchased from Sankyo Labo Service Corporation, Inc. (Tokyo, Japan). The rats were divided into four groups: control [normal-salt diet containing 0.3% NaCl, NS (−)], normal-salt diet with chaetocin [0.25 mg/kg of chaetocin (Sigma-Aldrich Co. LLC., St. Louis, MO, USA), NS (ch+)], HF [high-salt diet containing 8% NaCl, HS (−)], and treatment [high-salt diet with 0.25 mg/kg of chaetocin, HS (ch+)] group. Chaetocin was dissolved in dimethyl sulfoxide (DMSO). Rats in the treatment group were administered 0.25 mg/kg of chaetocin intraperitoneally twice a week from 6 weeks of age until 13 weeks of age, as previously reported^46^. Animals were sacrificed, and tissues were stored appropriately for individual experiments.

### Blood pressure and echocardiographic evaluations

Blood pressure levels were measured at 5, 8, 10, and 13 weeks using the tail-cuff method (BP-98 A-L; Softron, Tokyo, Japan). Hearts of 13-week-old rats were evaluated by echocardiography using a VisualSonics (Vevo 2100; VisualSonics Inc.). Detailed procedures for blood pressure measurements and echocardiographic evaluations are given in the Supplemental Methods.

### Morphometric analysis

Heart tissue was fixed in formalin, embedded in paraffin, and cut into 5-μm-thick sections. Sections were stained with hematoxylin-eosin or with EvG and picrosirius red to examine morphology and identify cardiac fibrosis. To evaluate collagen fiber quantity, the sections were observed using a fluorescence microscope (BZ-9000; KEYENCE Japan Inc. Osaka, Japan). The fibrotic volume fraction was assessed as the total fibrotic area (calculated by a cell-count system) divided by the total LV volume multiplied by 100.

### Quantitative real-time polymerase chain reaction (PCR)

Double-stranded cDNA was synthesized from 2 μg RNA and subjected to PCR with TaqMan Universal PCR Master Mix (Applied Biosystems, Foster City, CA, USA) and predesigned gene-specific primer and probe sets (TaqMan Gene Expression Assays: Applied Biosystems). Detailed procedures for real-time quantitative PCR are given in the Supplemental Methods.

### DNA microarray

RNA was extracted from LV samples in rats at the stage of heart failure using mirVana (Applied Biosystems), according to the manufacturer’s instructions. A mixture of RNA from three samples randomly chosen in each group was applied for genome-wide gene expression analysis using a Rat Oligo chip 20 K (TORAY). Gene ontology analysis was performed using GeneCodis3 (http://genecodis.cnb.csic.es/)^47^,^48^,^49^.

### ChIP

One gram of LV tissue per group was applied to ChIP with 25 μg antibody (targeting trimethylated K4 of histone H3 [#8580; Abcam, Tokyo, Japan] or trimethylated K9 of histone H3 [#07-442; Millipore, Temecula, CA, USA]), or 1 μg rabbit IgG (#2729; Cell Signaling Technology, Danvers, MA, USA). Detailed procedures for ChIP are given in the Supplemental Methods. In this paper, only the results of ChIP against trimethylated K9 of histone H3 are shown.

### Sequencing

Precipitated DNA fragment samples were further fragmented prior to sequence analysis (HiSeq 2000). Chromatin immunoprecipitated (ChIP’d) genomic DNA from each group and input DNA were sequenced according to the manufacturer’s instructions (Illumina). Additional details of the read alignments are given in the Supplemental Methods.

### Alteration of H3K9me3 level at the repetitive elements

We aligned the H3K9me3-ChIP’d reads to genomic features using both the rn4 assembly of the rat genome and a database of rat repetitive elements from the UCSC RepeatMasker track. If the ratio of nucleotide number overlapping between repetitive elements and sequenced reads to minimum number of nucleotides in the repetitive element or in the sequenced read was 90% or more, we considered it to be “overlapped”. We identified H3K9me3-enriched-regions on repetitive elements as those having 100-fold differences in H3K9me3 relative to input DNA.

### Isolation of heart mitochondria

Mitochondria were freshly isolated from 13-week-old hearts, as described previously^50^. Additional details are given in the Supplemental Methods.

### Mitochondrial oxygen consumption

Mitochondrial oxygen consumption (mitochondrial respiration) was measured with a Clark-type O_2_ electrode using the Oxygen Meter Model 781 and the Mitocell MT200 closed respiratory chamber (Strathkelvin Instruments) at 37 °C, as described previously^50^,^51^. Additional details are given in the Supplemental Methods.

### Mitochondrial protein and DNA content

The protein concentrations in mitochondrial fractions were measured using BCA protein assays. Mitochondrial DNA isolated from the heart was analyzed by real-time quantitative PCR using a Thermal Cycler Dice Real Time System TP800 (Takara Bio Inc., Shiga, Japan). All samples were normalized to genomic DNA content. Primer and probe sequences for each PCR are shown in Supplemental Table 2.

### Statistical analysis

The cumulative survival rates of rats in the different groups were compared using log-rank tests. All results are presented as means and standard deviations (SDs). Statistical comparisons were carried out using one-way analysis of variance (ANOVA) with post-Bonferroni corrections for Figs 1A–D and 4 and with post-hoc Dunnett corrections for Fig. 2B–D.

### Data deposition

Microarray data and ChIP-seq data are deposited in GEO with accession numbers GSE66617 and GSE69194.

## Additional Information

**How to cite this article**: Ono, T. *et al*. The histone 3 lysine 9 methyltransferase inhibitor chaetocin improves prognosis in a rat model of high salt diet-induced heart failure. *Sci. Rep.* **7**, 39752; doi: 10.1038/srep39752 (2017).

**Publisher's note:** Springer Nature remains neutral with regard to jurisdictional claims in published maps and institutional affiliations.

## Supplementary Material

Supplementary Information

## Figures and Tables

**Table 1:** DNA microarray analysis of LV tissue of DS rats.

Ensembl ID	RefSeq_ID	Symbol	Description	GO	Ratio	
				Mitochondrion	HS (−)/NS (−)	HS (ch+)/HS (−)
ENSRNOG00000025757	NM_017239.1	Myh6	Myosin-6 (myosin heavy chain 6) (MyHC-alpha)		0.2	1.59
ENSRNOG00000026548	XM_001078936.1;XM_213343.4	Dhrs7c	N/A		0.24	2.09
ENSRNOG00000012343	NM_145091.4	Pdp2	Pyruvate dehydrogenase (lipoamide)-phosphatase 2, mitochondrial precursor (PDP 2)	◯	0.27	1.89
ENSRNOG00000026679	NM_001008880.1	Scn4b	Sodium channel subunit beta-4 precursor		0.3	1.51
ENSRNOG00000006444	XM_001066628.1;XM_342763.3	Fkbp4	FK506-binding protein 4		0.32	1.6
ENSRNOG00000004078	NM_012949.2	Eno3	Beta-enolase (enolase 3)		0.33	1.77
ENSRNOG00000015904	NM_133581.1	Wfdc1	WAP four-disulfide core domain protein 1 precursor		0.34	1.59
ENSRNOG00000007290	NM_012505.1	Atp1a2	Sodium/potassium-transporting ATPase subunit alpha-2 precursor		0.34	1.45
ENSRNOG00000004377	NM_001012111.1	Lpin1	Lipin 1		0.34	1.46
ENSRNOG00000002827	NM_001106974.2	NP_001100444.2	Ataxin 2 binding protein 1		0.35	1.69
ENSRNOG00000021174	NM_139337.1	Lrp16	MACRO domain-containing protein 1 (protein LRP16)	◯	0.36	1.79
ENSRNOG00000018735	NM_013069.2	Cd74	H-2 class II histocompatibility antigen gamma chain (CD74 antigen)		0.36	1.85
ENSRNOG00000011260	NM_001008770.3	RGD:1306952	Carboxymethylenebutenolidase homolog (liver regeneration-related protein LRRG072)		0.37	1.54
ENSRNOG00000004640	NM_001006960.1	MGC94604	Mitochondrial protein, 18 kDa	◯	0.38	1.57
ENSRNOG00000000634	XM_001080360.1;XM_342127.3	RGD:1306739	N/A		0.39	1.81
ENSRNOG00000003977	NM_053769.3	Dusp1	Dual specificity protein phosphatase 1 (MKP-1)		0.4	1.54
ENSRNOG00000010697	NM_057186.1	Hadhsc	Hydroxyacyl-coenzyme A dehydrogenase, mitochondrial precursor	◯	0.41	1.4
ENSRNOG00000012091	XM_001075518.1;XM_227690.4	Ppa2	N/A	◯	0.41	1.96
ENSRNOG00000014641	XM_001058430.1;XM_213231.4	Rpl3l	N/A		0.41	1.66
ENSRNOG00000033924	XM_001077221.1	LOC691211	N/A		0.42	2.24
ENSRNOG00000014128	NM_001006986.1	MGC94704	Evolutionarily conserved signaling intermediate in Toll pathway, mitochondrial precursor	◯	0.43	1.71
ENSRNOG00000033615	N/A	NU3M_RAT	NADH-ubiquinone oxidoreductase chain 3		0.43	1.93
ENSRNOG00000002916	NM_019174.1	Ca4	Carbonic anhydrase 4 precursor		0.43	1.78
ENSRNOG00000039197	XM_001066530.1;XM_216399.4	Col15a1	Col15a1 protein		0.44	1.96
ENSRNOG00000015807	NM_001004261.1	RGD:1303232	Probable oxidoreductase C10orf33 homolog		0.45	1.47
ENSRNOG00000002272	NM_001108358.1	NP_001101828.1	Ligand of numb-protein X 1		0.45	1.65
ENSRNOG00000012827	NM_001107680.1	NP_001101150.1	Myeloid leukemia factor 1		0.45	1.7
ENSRNOG00000029613	XM_001074693.1;XM_223985.3	RGD:1562558	N/A		0.45	1.86
ENSRNOG00000002028	NM_001025014.1	Tmem50b	Transmembrane protein 50B		0.45	1.89
ENSRNOG00000021200	NM_001012080.1	Hfe2	Hemojuvelin precursor		0.45	1.43
ENSRNOG00000037446	NM_031587.1	Pxmp2	Peroxisomal membrane protein 2	◯	0.45	1.51
ENSRNOG00000021866	NM_001106601.1	NP_001100071.1	Similar to BolA domain-containing protein like (11.4 kD) (1P25)		0.46	1.93
ENSRNOG00000018516	NM_172224.1	Impa2	Inositol monophosphatase 2 (IMPase 2)		0.46	1.45
ENSRNOG00000013532	NM_017328.1	Pgam2	Phosphoglycerate mutase 2		0.47	1.45
ENSRNOG00000031321	XR_007906.1;XR_006141.1	LOC363987	N/A		0.47	1.78
ENSRNOG00000021812	XM_001060414.1;XM_001072661.1	LOC684826	N/A		0.48	1.48
ENSRNOG00000016369	NM_001006966.1	Peci	Peroxisomal delta3, delta2-enoyl-coenzyme A isomerase	◯	0.48	1.58
ENSRNOG00000006108	XM_001075781.1;XM_001081328.1	LOC690825	N/A		0.48	1.89
ENSRNOG00000016603	NM_201562.3	Rtn2	Reticulon 2 (Z-band associated protein)		0.48	1.58
ENSRNOG00000012249	XM_218658.4	Txlnb	Beta-taxilin (Muscle-derived protein 77)		0.48	1.69
ENSRNOG00000007069	NM_001025423.1	Adhfe1	Alcohol dehydrogenase, iron containing, 1	◯	0.48	1.46
ENSRNOG00000001912	NM_001108860.1	NP_001102330.1	Steroid 5 alpha-reductase 2-like 2		0.48	1.61
ENSRNOG00000017446	NM_001106322.1	NP_001099792.1	NADH dehydrogenase (ubiquinone) Fe-S protein 8	◯	0.49	1.54
ENSRNOG00000016368	NM_133425.3	Ppp1r14c	Protein phosphatase 1 regulatory subunit 14 C (PKC-potentiated PP1 inhibitory protein)		0.49	1.4
ENSRNOG00000018415	NM_001106111.1	NP_001099581.1	Thioesterase superfamily member 2	◯	0.49	1.46
ENSRNOG00000006968	NM_001029898.1	Mrpl19	Ribosomal protein, mitochondrial, L15	◯	0.49	1.41
ENSRNOG00000012455	NM_001011979.1	Tardbp	TAR DNA binding protein		0.49	1.86
ENSRNOG00000005987	NM_031127.3	Suox	Sulfite oxidase, mitochondrial precursor	◯	0.49	1.5
ENSRNOG00000005487	NM_001007750.1;XM_001079984.1	Chpt1	Cholinephosphotransferase 1 (diacylglycerol cholinephosphotransferase 1)		0.49	1.58
ENSRNOG00000015519	NM_001013889.1;NM_133295.3	Ces3	Carboxylesterase 3 precursor		0.49	1.84

GO: gene ontology; NS (−), normal salt diet without chaetocin; HS (−), high-salt diet without chaetocin; HS (ch+), high-salt diet with chaetocin.
