# Supplementary material for: The histone 3 lysine 9 methyltransferase inhibitor chaetocin improves prognosis in a rat model of high salt diet-induced heart failure
Source: Sci Rep. 2017 Jan 4;7:39752. doi: 10.1038/srep39752 (PMC5209701; doi:10.1038/srep39752)
Supplement: Supplementary Information [file srep39752-s1.pdf]

Supplementary Information

Manuscript title: **The histone 3 lysine 9 methyltransferase inhibitor chaetocin improves prognosis in a rat model of high salt diet-induced heart failure**

Authors:
Tomohiko Ono, Naomi Kamimura, Tomohiro Matsuhashi, Toshihiro Nagai, Takahiko Nishiyama, Jin Endo, Takako Hishiki, Tsuyoshi Nakanishi, Noriaki Shimizu, Hirotoshi Tanaka, Shigeo Ohta, Makoto Suematsu, Masaki Ieda, Motoaki Sano, Keiichi Fukuda, Ruri Kaneda.

Supplemental Methods

Blood pressure

Three blood pressure measurements of individual animals were recorded. Data are shown as mean ± SD of each group [the control group (n = 8), normal salt with chaetocin (n = 7), heart failure (HF) group (n = 9), or treatment group (n = 8)].

Echocardiographic evaluations

Hearts of 13-week-old rats in the control group (n = 9), normal salt with chaetocin (n = 6), HF group (n = 11), or treatment group (n = 16) were evaluated by echocardiography. Rats were anesthetized with 1-2% of isoflurane and their heart rates were kept around 300 beats per minutes. M-mode tracings were recorded through the anterior and posterior left ventricular (LV) walls at the papillary muscle level to measure anterior wall thickness (AWT), posterior wall thickness (PWT), LV end-diastolic diameter (LVEDD), and end-systolic diameter (LVESD), using a VisualSonics (Vevo 2100; VisualSonics Inc.) equipped with MS-250 imaging transducer. The following formulas were used to calculate LV fractional shortening (FS): FS (%) = [(LVEDD-LVESD)/LVEDD] × 100.

Real-time quantitative PCR

Total RNA was isolated by the acid-phenol extraction method (TRIzol, Invitrogen, Carlsbad, CA, USA) and subjected to isopropanol-ethanol precipitation to investigate the expression levels of *Nppa* and *Pgc1a* in accordance with the manufacturer’s protocol. To confirm the DNA microarray data of *Acadm*, *Ndufs4,* and *Coq3,* RNA was extracted with mirVana (Applied

Biosystems, Foster City, CA, USA). Double-stranded cDNA was synthesized from RNA (2 μg) using SuperScript II (Invitrogen) with oligo-dT primers and subjected to PCR with TaqMan Universal PCR Master Mix (Applied Biosystems). For the confirmation of DNA microarray data, a mixture of cDNA from the three samples applied to the microarray in each group was subjected to PCR. Detection of PCR products was monitored in real time with a 7500 real-time PCR system (Applied Biosystems), resulting in the calculation of a threshold cycle, or C_T value, defined as the PCR cycle number at which the exponential growth of the PCR product was observed. The C_T values for beta-actin (*Actb*) and the genes of interest (*Nppa, Pgc1a, Acadm, Ndufs4, and Coq3*) were used to calculate the abundance of the latter transcripts relative to *Actb* mRNA. Predesigned gene-specific primer and probe sets (TaqMan Gene Expression Assays: Applied Biosystems) were used and are described in Supplemental Table 1. All samples were run in triplicate.

Chromatin immunoprecipitation

One gram of crushed left ventricular tissue per group was cross-linked with 0.5% formaldehyde in D-PBS(-) (Wako, Osaka, Japan) for 10 min at 37℃, incubated in 125 mM glycine for 5 min at room temperature, resuspended in SDS lysis buffer (1% SDS, 10 mM EDTA, 50 mM Tris [pH 8.0]) supplemented with phosphatase inhibitor cocktail (Nacalai Tesque, Kyoto, Japan), and incubated on ice for 5 min. Samples were sheared to an average size of 500 bp by sonication of the lysate using a Bioruptor Ultrasonicator (Cosmo-Bio, Tokyo, Japan). Fragmented chromatin was incubated with Dynabeads M-280 sheep anti-rabbit IgG (DYNAL, Invitrogen) that had been pre-incubated with 25 μg of antibody (to trimethylated K4 of histone H3 [Abcam 8580] or trimethylated K9 of histone H3 [Millipore #07-442]), or with 1 μg of rabbit IgG (Cell Signaling Technology #2729) overnight at 4℃. The beads were then sequentially washed with Low-Salt Immune Complex Wash buffer (0.1% SDS, 1% Triton X-100, 2 mM EDTA, 20 mM Tris [pH 8.0], 150 mM NaCl), High-Salt Immune Complex Wash Buffer (0.1% SDS, 1% Triton X-100, 2 mM EDTA, 20 mM Tris [pH 8.0], 500 mM NaCl), LiCl Immune Complex Wash Buffer (0.5 M LiCl, 1% IGEPAL CA630, 1% deoxycholate, and 100 mM Tris [pH 7.5]), and Tris-EDTA buffer (10 mM Tris [pH 7.5], 0.1 mM EDTA). Protein-chromatin complexes were eluted with elution buffer (1% SDS, 0.1 M $NaHCO_3$). Cross-linking was removed in the eluted complexes by incubation in 200 mM NaCl at 65℃ for 6 hrs, after which proteins were digested with proteinase K at 55℃ for 2 hours and contaminant RNA fragments were removed with RNase A at 37℃ for 2 hours. Precipitated DNA fragments were purified by phenol chloroform-ethanol precipitation. (The data of ChIP-seq analysis for

K4me3 is not shown in this paper.)

Sequence read and alignment counts

Alignments of the reads were made with Bowtie (version 0.12.7) using the rn4 genome assembly of *Rattus norvegicus* from the UCSC genome browser as a reference sequence. Each run allowed for no more than three errors in 100 bp, and resultant alignments were converted to SAM format for further processing. This process yielded the following read and alignment counts:

- Input DNA quality-filtered reads, 77,593,626; reads aligned uniquely, 57,643,144
- K9me3-ChIP'd DNA of control group quality-filtered reads, 93,107,681; reads aligned uniquely, 73,356,636
- K9me3-ChIP'd DNA of HF group quality-filtered reads, 74,828,319; reads aligned uniquely, 58,645,109
- K9me3-ChIP'd DNA of treatment group quality-filtered reads, 65,994,592; reads aligned uniquely, 48,787,980

Isolation of heart mitochondria and assessment of mitochondrial function

Heart tissue was washed in ice-cold isolation buffer (225 mM mannitol, 75 mM sucrose, 5 mM HEPES, 1 mM EGTA) and minced with a razor blade. Trypsin was added to a concentration of 0.025% and the tissue further disrupted with a polytron tissue disruptor at moderate speed. After 10 min incubation on ice with intermittent gentle shaking, trypsin inhibitor (from soybean) was added to 0.25 mg/ml and the mixture homogenized with a motorized teflon/glass homogenizer. The homogenate was centrifuged for 5 min at 500 g. The supernatant was then centrifuged for 10 min at 8000 g at 4°C and the pellet suspended in isolation buffer. Unless noted otherwise, all procedures were performed on ice. Protein concentration was determined using the BCA protein assay.

Mitochondrial oxygen consumption

Mitochondria were incubated at 0.25 mg/ml in respiration buffer (125 mM KCl, 1 mM K_2HPO_4, 5 mM $MgCl_2$, 25 mM HEPES, 0.2 mM EGTA, 20 mM mannitol, 0.2% defatted BSA). Mitochondrial state 2 respiration was assayed with pyruvate/malate (each 2.5 mM) as substrates. State 3 and state 4 respiration was measured by adding ADP (0.25 mM) and oligomycin (2 µg / ml) to the assay buffer respectively.

Supplemental Table 1. TaqMan gene expression assays used for real-time quantitative PCR

Gene name	Symbol	Assay ID#
Natriuretic peptide precursor A	Nppa	Rn00561661_m1
Peroxisome proliferator-activated receptor gamma, coactivator 1 alpha	Pgc1a	Rn00580241_m1
Acyl-CoA dehydrogenase, C-4 to C-12 straight chain	Acadm	Rn00566390_m1
NADH dehydrogenase (ubiquinone) Fe-S protein 4	Ndufs4	Rn01489309_m1
Coenzyme Q3 methyltransferase	Coq3	Rn00569878_m1
Beta-actin	Actb	Rn00667869_m1

Supplemental Table 2. Primers and probes for real-time PCR of mitochondrial DNA

Mitochondrial DNA (cytochrome c oxidase 3 gene region)	
F primer	CGAGATATCATCCGTGAAGGAAC
R primer	GATTATTCCGTATCGGAGGCCT
Probe	ACCAAGGCCACCACACCCCTATTGTAC
Genomic DNA (chromosome 12, aldehyde dehydrogenase 2 gene region)	
F primer	CGCCCAAAAACCCAACAA
R primer	TTCTCTTGGCAGGCGCA
Probe	TCCTGAGAAAAGCCACCACCAAGCA

Supplemental Table 3. GO analysis by singular enrichment analysis of the cellular component (CC) category

Genes	NGR	TNGR	NG	TNG	Hyp	Hyp*	Annotations
13 genes	1247	29516	13	50	1.06E-07	8.79E-06	GO:0005739: mitochondrion (CC)
16 genes	3543	29516	16	50	0.000158607	0.00658218	GO:0005737: cytoplasm (CC)
1 gene	1	29516	1	50	0.001694	0.0281203	GO:0035692: macrophage migration inhibitory factor receptor complex (CC)
1 gene	1	29516	1	50	0.001694	0.0281203	GO:0019910: mitochondrial pyruvate dehydrogenase (lipoamide) phosphatase complex (CC)
1 gene	1	29516	1	50	0.001694	0.0281203	GO:0035693: NOS2-CD74 complex (CC)
2 genes	64	29516	2	50	0.00530207	0.0488968	GO:0005802: trans-Golgi network (CC)
2 genes	64	29516	2	50	0.00530207	0.0488968	GO:0042383: sarcolemma (CC)

NGR, number of annotated genes in the reference list; TNGR, total number of genes in the reference list; NG, number of annotated genes in the input list; TNG, total number of genes in the input list; Hyp, hypergeometric *p*-value; Hyp*, *p*-value calculated using the corrected hypergeometric distribution.

Supplemental Table 4. GO analysis by modular enrichment analysis of the biological process (BP), molecular function (MF), and cellular component (CC) categories

Genes	NGR	TNGR	NG	TNG	Hyp	Hyp*	Annotations
13 genes	1247	29516	13	50	1.06E-07	4.24E-06	GO:0005739: mitochondrion (CC)
3 genes	16	29516	3	50	2.52E-06	5.04E-05	GO:0005634: nucleus (CC)<br>GO:0030509: BMP signaling pathway (BP)
3 genes	41	29516	3	50	4.66E-05	0.000621257	GO:0005737: cytoplasm (CC)<br>GO:0006629: lipid metabolic process (BP)
7 genes	652	29516	7	50	0.000108826	0.00108826	GO:0016787: hydrolase activity (MF)
16 genes	3543	29516	16	50	0.000158607	0.00126885	GO:0005737: cytoplasm (CC)
3 genes	75	29516	3	50	0.000283429	0.00188953	GO:0005829: cytosol (CC)<br>GO:0016787: hydrolase activity (MF)
3 genes	85	29516	3	50	0.000409672	0.00234098	GO:0016853: isomerase activity (MF)
5 genes	483	29516	5	50	0.0013283	0.00590356	GO:0016491: oxidoreductase activity (MF)
3 genes	125	29516	3	50	0.00125675	0.00628376	GO:0005739: mitochondrion (CC)<br>GO:0016491: oxidoreductase activity (MF)
3 genes	140	29516	3	50	0.00173902	0.00695608	GO:0000287: magnesium ion binding (MF)
3 genes	153	29516	3	50	0.00223924	0.00814268	GO:0009055: electron carrier activity (MF)

NGR, number of annotated genes in the reference list; TNGR, total number of genes in the reference list; NG, number of annotated genes in the input list; TNG, total number of genes in the input list; Hyp, hypergeometric *p*-value; Hyp*, *p*-value calculated using the corrected hypergeometric distribution. The annotations with $p < 0.01$ (Hyp*) are listed.

Supplemental figure 1

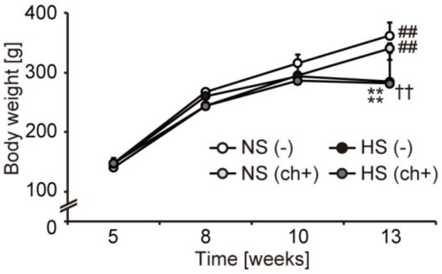

Supplemental figure 2

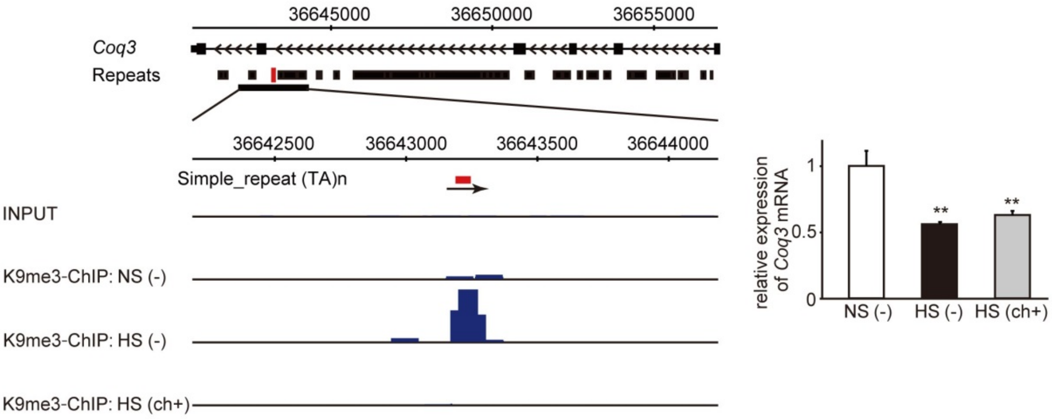

Supplemental Figure Legends

Supplemental Figure 1. Time course of changes in body weight.

Body weights in the control group [NS (-), n = 15], normal salt diet with chaetocin group [NS (ch+), n = 12], HF group [HS (-), n = 36], and treatment group [HS (ch+), n = 32]. Data are presented as the mean ± SD. Statistical comparisons at 13 weeks of age were examined by one-way ANOVA with post-hoc Bonferroni correction. $^{**}P < 0.01$ versus the control group; $^{\#\#}P < 0.01$ versus the HF group; $^{\dagger\dagger}P < 0.01$ versus the normal salt diet with chaetocin group.

Supplemental Figure 2. Enrichment of H3K9me3 repetitive loci in regions in close proximity to *Coq3*

The red square indicates the region that was identified as being enriched in H3K9me3 repetitive elements in rats with heart failure. The black boxes indicate repetitive loci. The blue bars indicate H3K9me3 read alignments. *Coq3* mRNA expression was determined using real-time quantitative PCR and is shown as the fold change versus that in the control group. $^{**}P < 0.01$ versus the control group. Statistical comparisons were carried out using one-way analysis of variance (ANOVA) with post-hoc Bonferroni corrections.
